# Early choroidal and retinal changes detected by swept-source oct in type 2 diabetes and their association with diabetic kidney disease: a longitudinal prospective study

**DOI:** 10.1186/s12886-024-03346-4

**Published:** 2024-02-23

**Authors:** Monica Oliveira da Silva, Anne Elise Cruz do Carmo Chaves, Glauber Corrêa Gobbato, Fabio Lavinsky, Daniel Lavinsky

**Affiliations:** 1 Department of Ophthalmology, Hospital de Clínicas de Porto Alegre, Federal University of Rio Grande do Sul, UFRGS, Porto Alegre, Brazil; 2 Retina and Vitreous Research Center, Hospital de Clínicas de Porto Alegre, Porto Alegre, Brazil; 3 Graduate Program in Endocrinology, Federal University of Rio Grande do Sul, UFRGS, Rua Landel de Moura 550/209, Porto Alegre, RS 91920-150 Brazil; 4 Medical School, UNISINOS University, Porto Alegre, Brazil; 5 Lutheran University of Brazil Medical School, Porto Alegre, Brazil

**Keywords:** Diabetes mellitus type 2, Diabetic nephropathy, Diabetic retinopathy, Retinal thickness, Choroidal thickness

## Abstract

**Background:**

To evaluate structural changes in retina and choroid in patients with type 2 diabetes (T2D) and their association with diabetic kidney disease (DKD).

**Methods:**

T2D patients with mild or no diabetic retinopathy (DR) were followed for 3 years using structural SS-OCT and OCT angiography (OCT-A) taken every 6 months. Parameters were compared longitudinally and according to the DKD status on baseline.

**Results:**

One hundred and sixty eyes from 80 patients were followed for 3 years, 72 with no DKD (nDKD) at baseline and 88 with DKD. Trend analysis of T2D showed significant thinning in GCL + and circumpapillary retinal fiber neural layer (cRFNL), choroid, and decreased vascular density (VD) in superficial plexus and central choriocapillaris with foveal avascular zone (FAZ) enlargement. Patients with no DKD on baseline presented more significant declines in retinal center and choroidal thickness, increased FAZ and loss of nasal and temporal choriocapillaris volume. In addition, the nDKD group had worse glycemic control and renal parameters at the end of the study.

**Conclusion:**

Our data suggests the potential existence of early and progressive neurovascular damage in the retina and choroid of patients with Type 2 Diabetes (T2D) who have either no or mild Diabetic Retinopathy (DR). The progression of neurovascular damage appears to be correlated with parameters related to glycemic control and renal damage.

## Background

Diabetes mellitus (DM) stands as a paramount global public health issue, marked by hyperglycemia arising from impaired insulin production and distinct altered metabolic pathways. These pathways contribute to oxidative stress, a key factor in the pathophysiology of diabetic retinopathy (DR) and other chronic complications associated with all forms of diabetes mellitus, as Diabetic kidney disease (DKD) [1, 2].

The congruence in structural characteristics and analogous metabolic pathways observed between the glomerular blood filtration barrier, and the inner blood-retinal barrier (BRB), has instigated scientific investigation into the interconnection between the two microvascular disorders, DKD and DR [3, 4].

The clinical categorization of DR relies on fundus examination, encompassing the identification of microaneurysms, hemorrhages, and vascular changes, including venous beading, microaneurysms, intraretinal microvascular abnormalities (IRMA), neovascularization [5].

The optical coherence tomography (OCT), an advanced technology capable of producing detailed retina images, currently does not contribute to this classification, however it is capable to identify early changes in retinal vascular morphology in patients with little or no retinal damage and, additionally, provides indications of early retinal neurodegeneration.

Retinal vascular cells (endothelial cells and pericytes) forms with retinal neural cells (such as ganglion, amacrine, bipolar and horizontal cells) and glia a functional and structural complex: the neurovascular unit (NVU). This complex is highly susceptible to oxidative and metabolic damage and [6] There is empirical evidence suggesting early neurodegeneration, retinal vascular damage and choroidal changes in diabetic patients with no clinical signs, from previous studies using OCT, most of them with Spectral Domain technology (SD-OCT) [7].

The Swept-source OCT (SS-OCT), a novel OCT technology, has enhanced image penetration through the utilization of a longer laser wavelength (1050 nm). As result, SS-OCT high-resolution images allow a detailed information on the vascular architecture of the posterior pole, retinal and choroidal layers [8].

Furthermore, SS-OCT dye-free angiography (OCT-A) enables to asses reliable data derivated from depth imaging of structural retinal and choroidal vasculature, characterized by high resolution and a significant decrease in motion artifacts [9].

Despite indications pointing towards neuroglial impairments and blood flow abnormalities occurring prior the onset of DR, the evolution of these findings over time until the appearance of clinical signs, remains unclear. Therefore, this study aimed to identify and monitor early neurovascular changes in the retina and choroid in patients with type 2 diabetes (T2D), comparing groups with or without DKD, utilizing SS-OCT and OCT-A, over a 3-year follow-up period.

## Methods

### Settings and participants

**Fig. 1:**
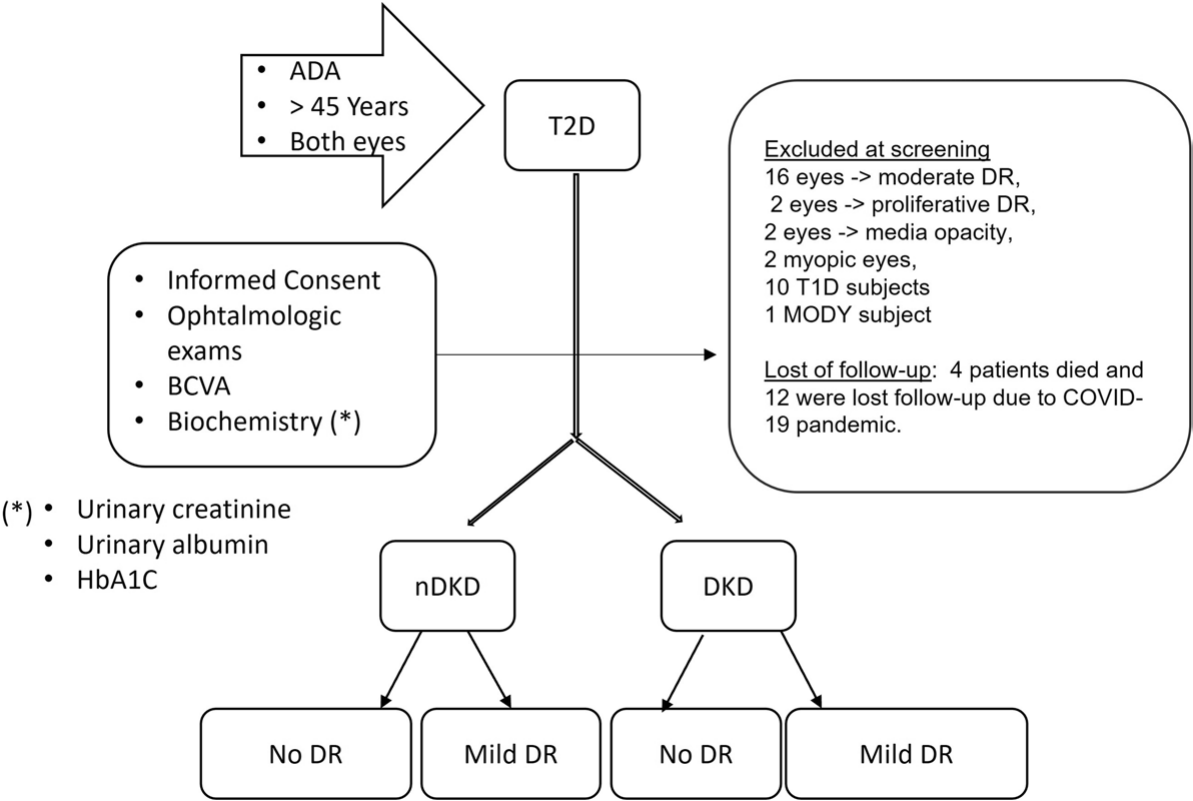
Flowchart screening visit

This longitudinal study was conducted at a Hospital de Clinicas de Porto Alegre (HCPA) from July 2018 to February 2022. The study protocol adhered to the principles of the Helsinki Declaration and obtained approval from the HCPA Ethics Committee. Patients were allocated in systematic and consecutive enrollment of cases. At screening visit, after performed informed consent procedures, were collected demographic information, medical history, and biological samples of glycosylated hemoglobin (HbA1C), urinary creatinine and albumin excretion (UAE). Ophthalmologic examination included functional evaluation with best corrected visual acuity (BCVA), and structural analysis using SS-OCT and OCT-A (Fig. 1).

The inclusion criteria allowed inclusion of both eyes of T2D patients (American Diabetes Association criteria), aged ≥ 45 years with no previous bariatric surgery [10]. Eyes presenting at least one of the following characteristics at baseline were excluded: Lens opacities, glaucoma, previous surgery, spherical equivalent exceeding ± 3 diopters, DR moderate or severe, or central macular edema.

Visual Acuity (BCVA and SS-OCT/OCT-A were performed every three months over the three-year follow-up period. Kidney function, measured by UAE and eGFR, as well as glycemic control, assessed by HbA1c levels, were re-evaluated at months 12, 24, and 36.

### SS-OCT and OCT-A imaging

Swept source OCT and OCT-A images protocol follow the same described in previous analysis [11, 12]. All examinations were performed every 6 months. and carried out in the morning (9:00–11:00 h a.m.) to reduce effects of diurnal variations. Diabetic retinopathy was graded by a masked ophthalmologist based on the international clinical diabetic retinopathy and diabetic macular edema disease severity scales [5].

### Diabetic kidney disease

Diabetic kidney disease (DKD) was characterized by the presence of urinary albumin excretion (UAE) exceeding 14 mg/L and/or a reduced estimated glomerular filtration rate (eGFR < 60 mL/min/1.73 m2) [13, 14]. The eGFR was determined using the CKD-EPI (Chronic Kidney Disease Epidemiology Collaboration) equation.

## Statistics

Statistical data were analyzed using IBM SPSS software (version 26.0). The Shapiro–Wilk test was used to evaluate normal distribution. Comparisons between groups were performed using a chi-square test for nominal variables and a two-samples independent t-test for continuous variables. Given the potential inclusion of both eyes, generalized estimating equations (GEE) were employed to evaluate intergroup variances, adjusted by age; missing values excluded. Rates of changes were calculated with mixed effect models. Urinary albumin was analyzed using gamma distribution. Relationships between pairs of continuous variables were verified by Spearman correlation analysis. The sample size considered a difference of 15 μm in the choroid and 40 μm in the retina with a power of 80%, considering an α error of 0.05 and β = 0,20; all tests were two-tailed.

## Results

Demographic and clinical features of the 160 eyes that met inclusion criteria are presented in Table 1: 72 eyes of 36 subjects with no DKD (nDKD) and 88 eyes of 44 subjects with DKD. We excluded 16 eyes due to moderate DR, 2 eyes with proliferative DR, 2 eyes with media opacity, 2 myopic eyes, 10 patients with T1D, and 2 with MODY. During follow-up, four patients died and 12 were lost follow-up due to COVID-19 pandemic.

**Table 1:** Baseline demographic and clinical characteristics of patients with no or mild diabetic kidney disease (nDKD) and diabetic kidney disease (DKD) vs. controls

Variable Baseline (*n* = 160 eyes of 80 study participants	nDKD (*n* = 72 eyes of 36 subjects)	DKD (*n* = 88 eyes of 44 subjects)	*P*-value
Gender, n (%)^a^			
Male	12 (33.3%)	19 (43.2%)	0.203
Female	24 (66.7%)	25 (56.8%)	
Age in years, mean (SD)^b^	60.3 (± 7.6)	59.7 (± 7.8)	0.581
Ethnicity, n (%)^a^			
Caucasian	66 (91.7%)	81 (92.0%)	0.931
African descent / mixed	6 (8.3%)	7 (8.0%)	
Diabetic retinopathy stage, n (%)^b^			
Without DR	66 (91.7%)	62 (70.5%)	**0.002**
Mild NPDR	6 (8.3%)	26 (29.5%)	
DM duration in years, mean (SD)^b^	13.2 (± 7.8)	13.9 (± 6.4)	0.524
HbA1c, mean (SD)	7.8% (± 1.4)	8.6% (± 1.7)	**0.002**
BCVA, mean (SD)	47.8 (± 1.3)	45.0 (± 1.5)	0.366
Hypertension, n (%)	60 (88.2%)	80 (90.9%)	0,585
UAE (SD)^b^	4.3 (3.0–9.6)	30 (19.4–124.5)	**0.040**
eGFR (SD)	88.6 (± 0.6)	78.1 (± 3.8)	**0.008**

Significant values in bold

*BCVA* Best corrected visual acuity (number of letters read), *UAE* Urinary albumin excretion, *eGFR* Estimated glomerular filtration rate

^a^Subjects

^b^Median

### Baseline sample: demographic and clinical characteristics

Our population was predominantly composed of Caucasian females, but gender and ethnicity, as well as age, DM duration and prevalence of hypertension were comparable between the nDKD and DKD groups at baseline (BL). Mean age was 60 years, and the average duration of DM was 13.5 years. Patients with DKD at BL showed higher prevalence of mild DR (29.5% vs. 9.7%; *P* = 0.002) and higher HbA1c than nDKD (8.6% vs. 7.8%, *P* = 0.016). The median UAE nDKD was 4.3 mg/L (3-9.6) and 30 mg/l (19.4–124.5) among nDKD patients and 30 mg/l in the DKD group (95% CI -334.9 to -7.8; *P* = 0.040). Structural OCT/OCT-A parameters had no statistical difference between nDKD and DKD groups on baseline.

### Clinical changes in 3 years study follow-up

T2D patients were followed during 3 years in a mean of 7 follow-up visits.

All patients, both DKD and no-DKD, were under the care of a public endocrinology health unit and followed international protocols for the indication of insulin and/or metformin use. Endocrinologists monitored and adjusted medications throughout the study to optimize glycemic control based on individual patient needs.

Individuals underwent treatments for DM management based on their individual health support. Throughout the 3-year follow-up period, only one patient reported undergoing DME treatment in the last year, specifically with focal laser. The remaining participants did not indicate initiating any treatment during the study duration.

Kidney function were followed according to status (nDKD and DKD) at BL. In the nDKD group, 6 eyes (8.3%) had mild DR at BL and 1 eye progressed to moderate DR (16.7%). Seven eyes (10.6%) progressed from no DR to mild DR, 2 of which also progressed to macular edema, and 2 (3.0%) eyes from mild to moderate. In the DKD group, 24 eyes (29.5%) presented mild DR at BL, of which 6 eyes (23.1%) progressed from mild to moderate and 4 eyes (15.4%) regressed to no DR. Among those without DR at BL, 8 eyes (12.9%) progressed to mild DR (2 with macular edema), 2 (3.2%) to moderate and 2 (3.2%) to proliferative plus macular edema. Two subjects in this group underwent bariatric surgery during study follow up and showed improvement of renal function from DKD to nDKD.

The nDKD and DKD groups showed no significant BCVA changes over time (respectively: 95% CI -4.9 to 1.8; *P* = 0.366; 95% CI -3.6 to 2.5, *P* = 0.715). Nevertheless, DKD patients had worse visual acuity at the end of the study (44.5 ± 1.7 vs. 49.4 ± 1.3; 95% CI -9.3 to -0.5; *P* = 0.029). Although Hb1Ac was significantly different between the DKD and nDKD groups at baseline, no significant difference was observed between the groups at the end of follow up period, with worsening in the nDKD values (95% CI 0.6 to 1.7, *P* < 0.001; CI -8.6 to -1.1; *P* = 0.012), that showed a mean of 8.6% vs. 8.4% (*P* = 0.343).

Estimate glomerular filtration rate values also showed worsening in the nDKD group (95% CI 0.6 to 1.7, *P* < 0.001; CI -8.6 to -1.1; *P* = 0.012). Albuminuria (UAE) increased in both groups by the end of study: median 12.1 mg/L (5.6–19.1) (95% CI 3.1 to 34.3; *P* = 0.019) in the nDKD group and 57.2 mg/L (26.5–211.0) (95% CI 5.0 to 583.8; *P* = 0.046) in the DKD group. The difference between groups remained significant from baseline through the end of the study (95%CI -804.0 to -90.2; *P* = 0.014).

### Retinal layers

Table 2 shows the progression of retinal and choroidal parameters over study follow-up. Retinal center thickness (RCT) showed no significant changes (95% CI -4.9 to 2.9; *P* = 0.604). Ganglion cell layer plus (GCL+) and circumpapillary retinal fiber layer (cRNFL) became thinner in almost all quadrants. Total GCL + thinning in a rate of change of -0.3 μm ± 0.1 μm/year. Diabetic patients presented thinning of cRNFL but renal function had no effect on cRNFL changes over time, and both the nDKD and DKD groups showed the same trend towards decreased thickness.

**Table 2:**
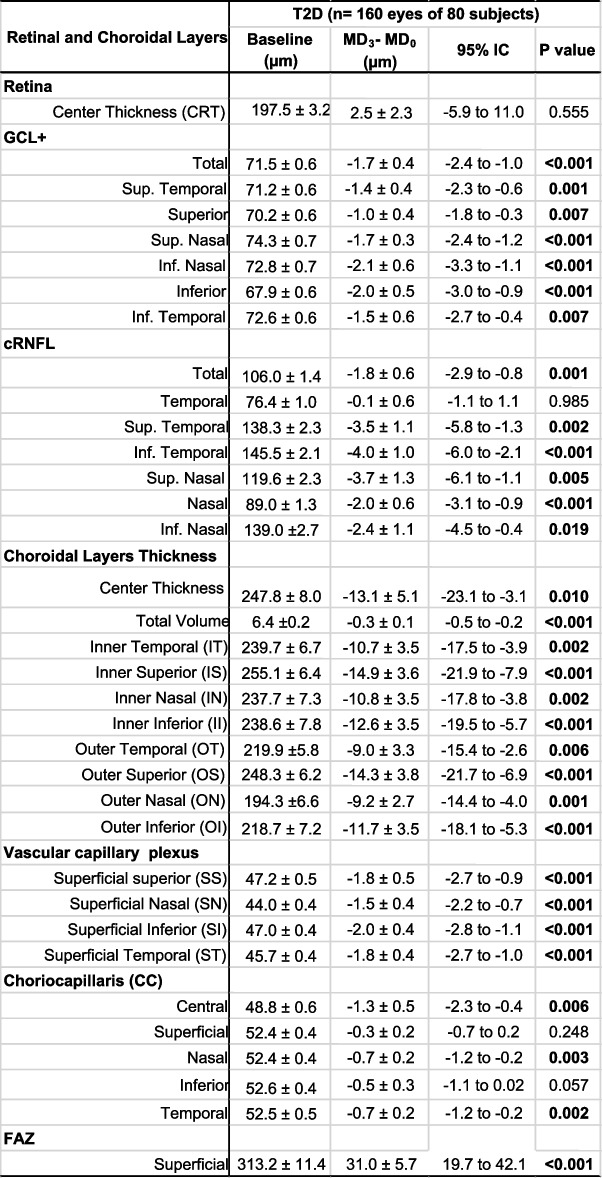
Retinal and choroidal layers thickness and density in T2D patients

All values are given as mean ± SD. Significant values in bold

*GCL+* Ganglion cell layer plus, *RNFL* Retinal nerve fiber layer, *FAZ* Foveal avascular zone

 RCT showed a slight decrease in the nDKD group (95% CI -12.0 to -0.1; *P* = 0.047) during follow-up; however, the DKD group remained without significant changes (Table 3). Decrease of GLC + thickness was observed only in the nDKD group and in the following quadrants: superior (*P* = 0.040), superior temporal (*P* = 0.033), superior nasal (*p* = 0.027), and GCL + total (*P* = 0.005) (Table 3).

**Table 3:**
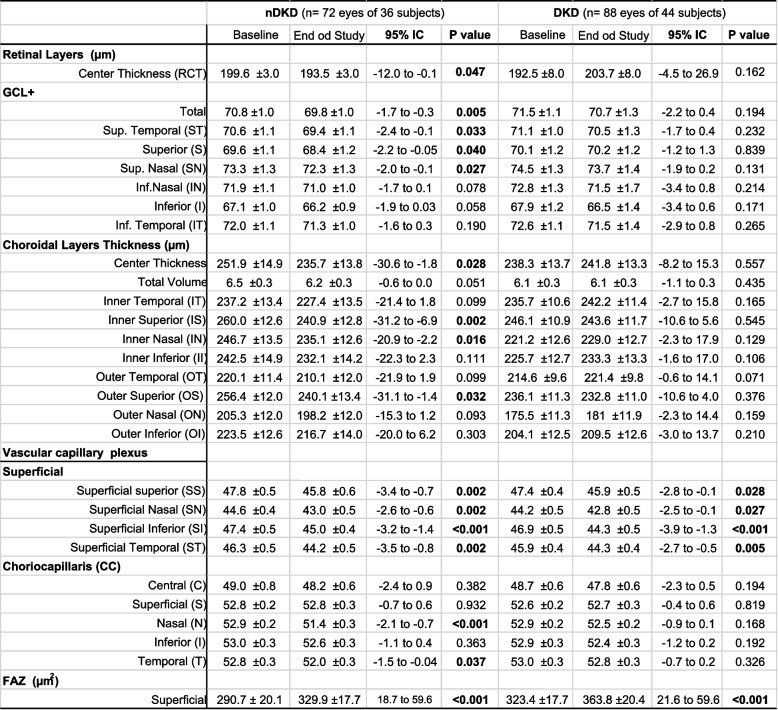
Choroidal thickness, retinal layers thickness and vascular capillary plexus in nDKD and DKD groups

*GCL+* Ganglion cell layer plus

### Choroidal layers

All choroidal layers, both in ETDRS inner and outer rings, showed thinning over study time in T2D patients (Table 2). When we look at kidney function effect, nDKD group showed significant decreased in quadrants inner and outer superior (*P* = 0.002 and *P* = 0.032, respectively), nasal (*P* = 0.016) and center thickness (*P* = 0.028) (Table 3).

### OCT-A

**Fig. 2:**
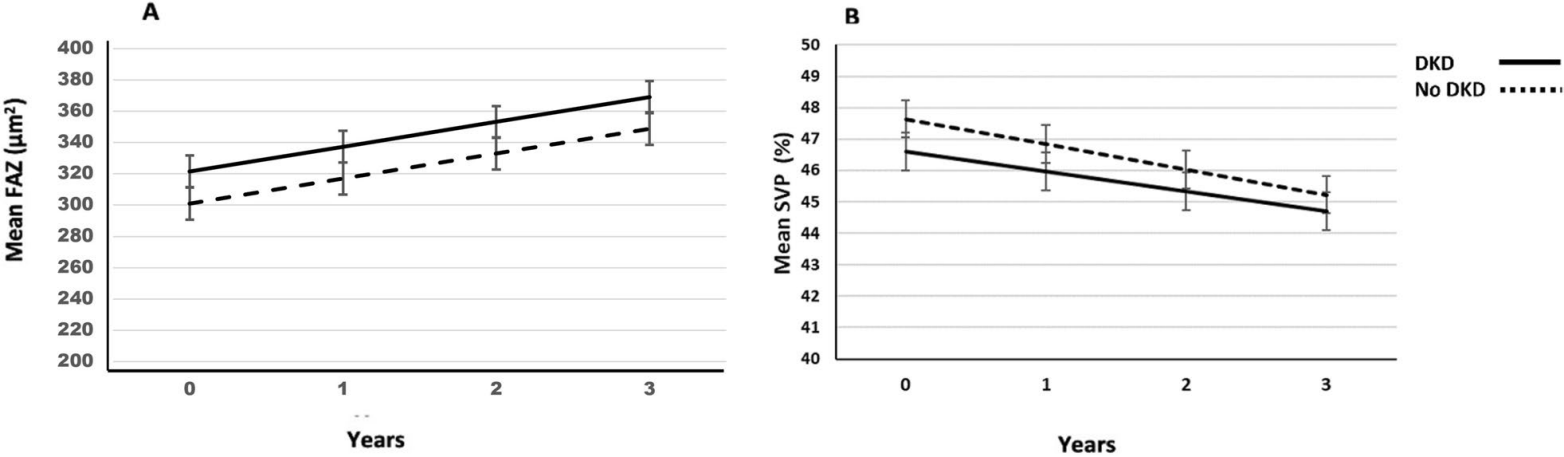
**A** FAZ mean rate of change 15.8 ± 2.9 µm^2^/year, 95% CI 10.1 to 21.5, *P* < 0.001; **B** PVS retina, inferior quadrant, mean rate of change − 0.6 ± 0.2 μm/year, 95% CI -1.0 to -0.3, *P* = 0.001

OCT-A showed decrease of retina vascular density in superficial plexus to all ETDRS quadrants and a significant increase of fovel avascular zone (FAZ) over time (*P* < 0.001) (Fig. 2). Similar results were observed in nDKD and DKD groups (Table 3). Choriocapillaris of T2D showed significant decrease of vascular density in central (*P* = 0.006), nasal (*P* = 0.003) and temporal quadrants (*P* = 0.002), but only nDKD group registered these changes in nasal (*P* < 0.001) and temporal (*P* = 0.037) quadrants.

## Discussion

This was a prospective longitudinal study that followed 160 eyes from 80 T2D patients with no or mild DR for 3 years, evaluated the trend of changes in retinal and choroidal layers changes and compared the impact of renal function in neurovascular damage over time.

We found a significant and progressive thinning of cRNFL and GCL + layers, decreased superficial vascular plexus (SVP) density and enlargement of FAZ area, indicating progressive neurovascular damage in this population of patients with T2D. Retinal center thickness did not show thickening during follow-up, suggesting that these changes were not associated with center-involving macular edema.

The retinal vasculature supports neural function, interacting with neurons and glia in the NVU to maintain the environmental homeostasis, necessary to preserve retinal structure and function. Microvascular damage is expected to be one of the earliest manifestations of NVU disruption; nevertheless, Santos et al. (2017) found that 61% of patients without microvascular disease presented evidence of neurodegeneration, leading the authors to conclude that neurodegeneration plays a role in the pathogenesis of DR but not in all patients with T2D [15]. In our study, we found that early vasculopathy and neurodegeneration occurred concurrently, which may be explained by the duration of DM at baseline. Aschauer et al. observed similar results in a 2-year follow-up of T2D patients who presented with microangiopathy and neurodegeneration, appearing progressively and concomitantly in the early stages of DR [16].

In our study we found early vasculopathy and neurodegeneration concurrently. Nevertheless, Leung et al. (2013) using a Cirrus HD-OCT showed that GCL + decreases approximately a rate of -0.32 μm/year in a healthy sample with mean of age 58.15 ± 6.84 years [17]. Lim et al. also found in their study a GCL + progression of -0,28 μm/year in the healthy control group [18]. These values are consistent with the results we found to GCL + rate of progression, suggesting that the trend of thickness observed may be attributed to ageing effect.

The effect of age is not sufficient, by itself, to explain the trend to decrease vascular density and increased FAZ area that we observed in OCT-A. Using a Spectral domain OCT-A to evaluate a healthy population age 41.3 years, Lavia et al. found a mean increase of FAZ area of 3.0 µm^2^/year and decrease of SVP in 0.06%/year [19]. Aschauer et al., in a study with T2D patients with a mean age of 57 ± 10 years, found enlargement of FAZ in a rate of 8.0 ± 2.0 3µm^2^/year [16]. Our T2D patients showed a FAZ rate of progression of 15.7 µm^2^/year and between − 1.3 to -0.9% the rate of thinning in SVP, observed both in nDKD as DKD groups. These results are consistent with progressive, degenerative effect of DM on inner retina micro vessels and suggests an early and clear vascular damage in inner retina that can lead to an impact on NVU in these early stages of DR.

The microvascular complications of DM, which include DR and DKD, share some common risk factors, such as poor glycemic control and DM duration; this has led authors to investigate – and find associations – between albuminuria and DR, even in its early stages [20]. A recent meta-analysis investigating the association between retinal microvascular signs and kidney disease found that DR was associated with CKD in T2D patients [21]. Previous studies also showed that microalbuminuria, an early marker of nephropathy and endothelial dysfunction, and eGFR are clinically significant risk factors for DR [22, 23].

Our population suffered the consequences of the COVID-19 pandemic. This might be one of the reasons of significant worsening of glycemic levels and kidney function in the nDKD group during follow-up, and the metabolic impact of these changes may explain their worse retinal, choroidal, and renal biomarker findings. Retinal center thickness (RCT) that may be related to worse glycemic control, whose metabolic impact also could be related to results of kidney biomarkers at the end of study.

Nevertheless, the negative effect of DKD on baseline was observed on visual acuity parameter. A worse BCVA after 3 years follow up in the DKD group may be associated with loss of VD. This find is consistent with Ghassemi et al., that consider parafoveal VD of retinal superficial plexus and subfoveal CC as a biomarker to predict visual acuity [24].

Discrepant results have been reported regarding choroidal parameters, such as thickness and volume, in recent studies of both T1D and T2D patients. Malerbi et al. (2018) reported increased subfoveal choroid thickness in T1D with microalbuminuria vs. normal UAE [22]. Oliveira-Ferreira et al. (2020) also found thicker subfoveal and temporal CT in T2D patients with microalbuminuria compared to diabetics with normal UAE and healthy controls [25]. Increase of CT in the early stages followed by decrease were observed both in T1D as T2D patients [26, 27].

Decrease of CT, even in early stages, has been shown in diabetic patients, mainly in patients with decrease UAE [28, 29]. A recent meta-analysis also found thinner subfoveal CT in diabetic patients compared to controls [30]. In our sample we found a significant trend of thinning in choroid of T2D patients, also observed in nDKD group. Liu et al. (2020) suggested that these conflicting results could be explained by different protocol designs, patient profiles, adjustment for confounding factors, and different protocol devices [31].

The strength of our study is the longitudinal design to follow the changes in retinal and choroidal layer density and thickness for 3 years, using SS-OCT technology, which enables more precise identification of the choroidal-scleral edge and, consequently, more reliable CT and CC density measurements. The main limitations were the absence of a healthy control group, relatively small sample size, population with a high prevalence of white women and no age- matched controls. The initial study protocol incorporated the screening of a health control group, pivotal in the cross-sectional primary analysis [11, 12]. Yet, in response to the imperative of ensuring health safety amid the COVID-19 pandemic, we opted to abstain from enlisting health volunteers in the study. Conversely, routine eye examinations remained of significance for individuals with T2D.

Diabetic patients without clinical signs of DR represents a great therapeutic opportunity to preserve vision. Longer follow-up is recommended to observe the structural changes and DR progression in eyes of T2D patients associated with kidney parameters.

In conclusion, our study shows progressive neurovascular damage in eyes of T2D patients with no or mild DR, with significative effects on SVP density and enlargement of FAZ. Results also suggest a possible association between worsening glycemic control and renal parameters with increased trend of neurovascular (structural) damage in T2D patients with no DKD and no DR, while functional decline, as evidenced by BCVA, was observed in patients with DKD in comparison to those without DKD.

## Data Availability

Patient data are registered in medical records and on the OCT device. According to Brazilian legislation, data can only be accessed with express permission from HCPA Ethical Committee, patients or their legal representatives. To obtain permission to access patient and research data, should be contact the following investigator: Mônica Oliveira da Silva, e-mail cebio.monica@gmail.com, address Ramiro Barcelos 2350 Ophthalmology Unit Zip Code 90035-003, Porto Alegre, RS, Brazil.

## Abbreviations

BCVA: Best corrected visual acuity
BL: Baseline
BM: Bruch’s membrane
BRB: Blood-retinal barrier
CC: Choriocapillaris
cRNFL: Circumpapillary retinal fiber layer
DKD: Diabetic kidney disease
DM: Diabetes mellitus
DR: Diabetic retinopathy
eGFR: Estimated glomerular filtration rate
ETDRS: Early Treatment Diagnostic Retinopathy Study
FAZ: Foveal avascular zone
GCL+: Ganglion cell layer plus
GEE: Generalized estimating equations
HbA1C: Glycosylated hemoglobin
IRMA: Intraretinal microvascular abnormalities
NVU: Neurovascular unit
OCT: Optical coherence tomography
OCT: A-OCT angiography
RCT: Retinal center thickness
RCT: Retinal center thickness
SD: OCT-Spectral Domain technology
SD: Standard deviation (SD)
SS: OCT-Swept-source OCT
SVP: Superficial vascular plexus
T2D: Type 2 diabetes
UAE: Urinary albumin excretion
